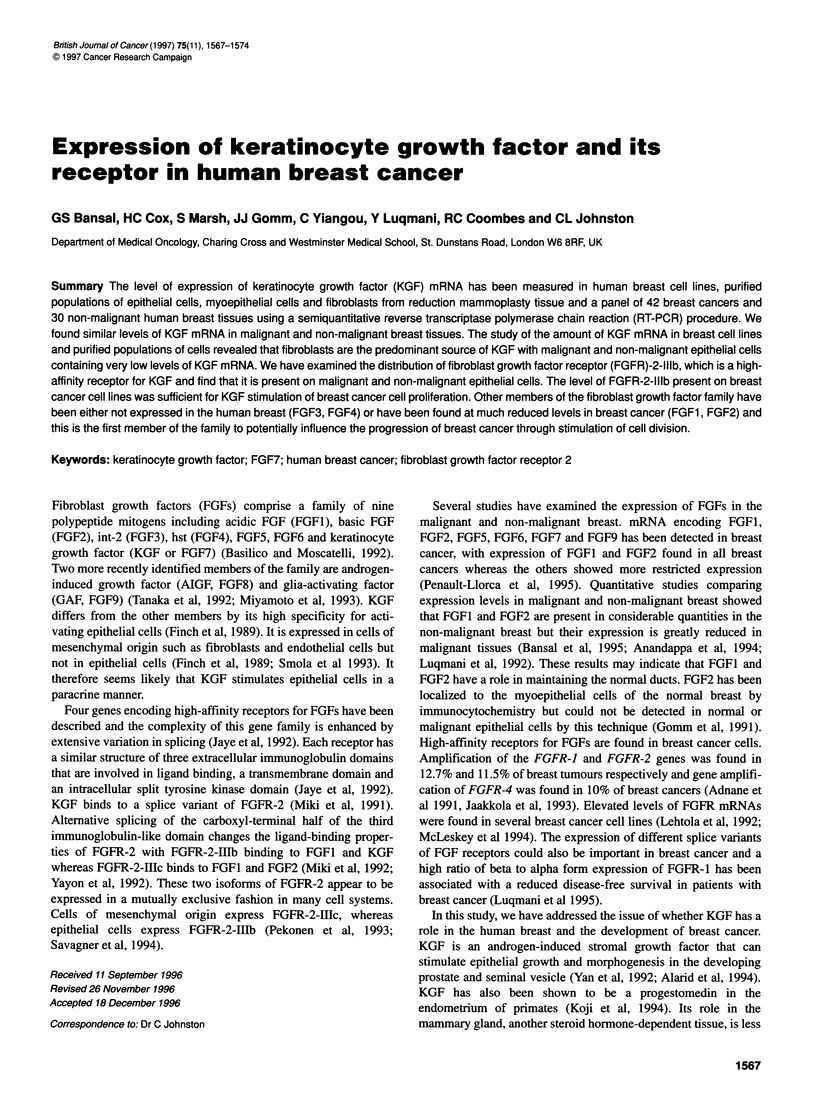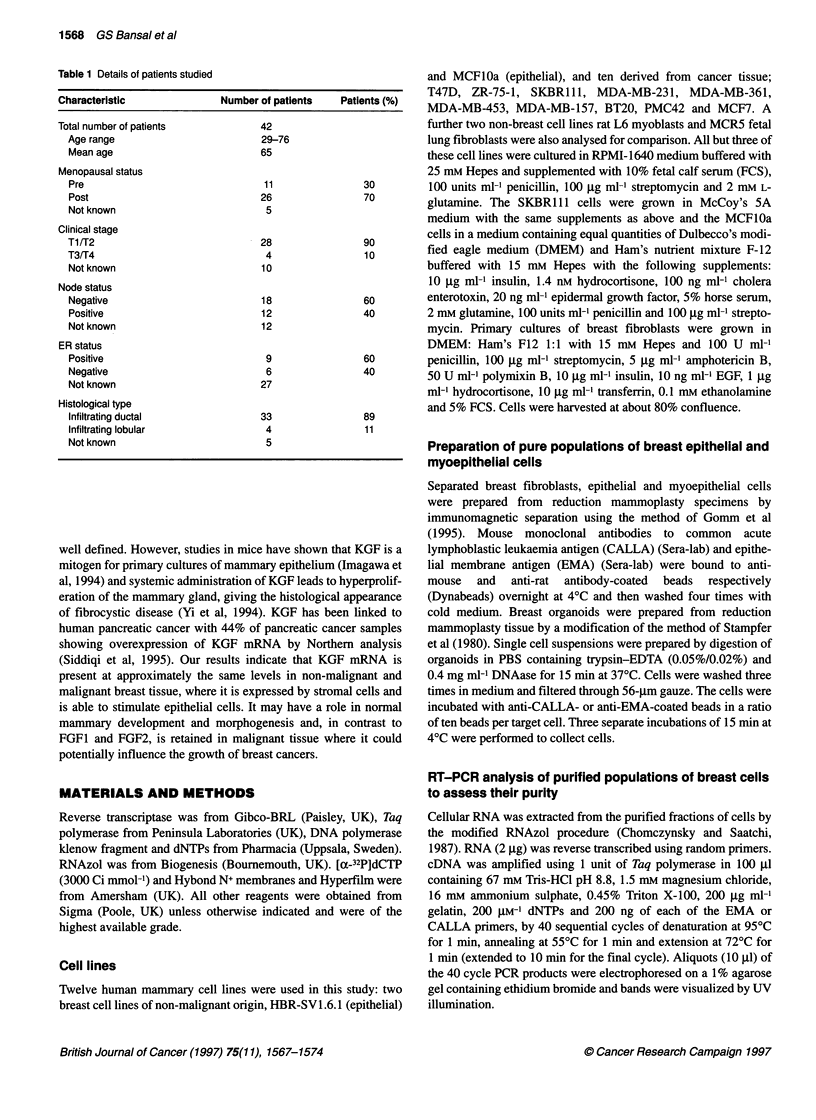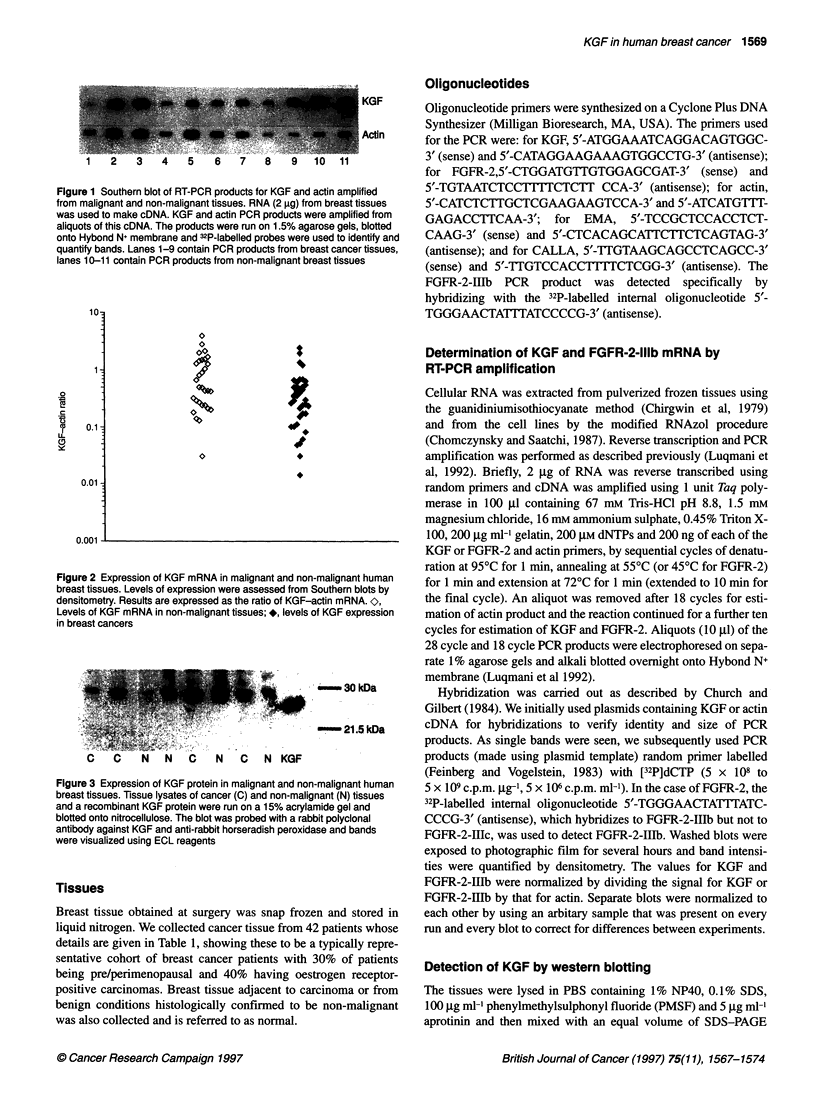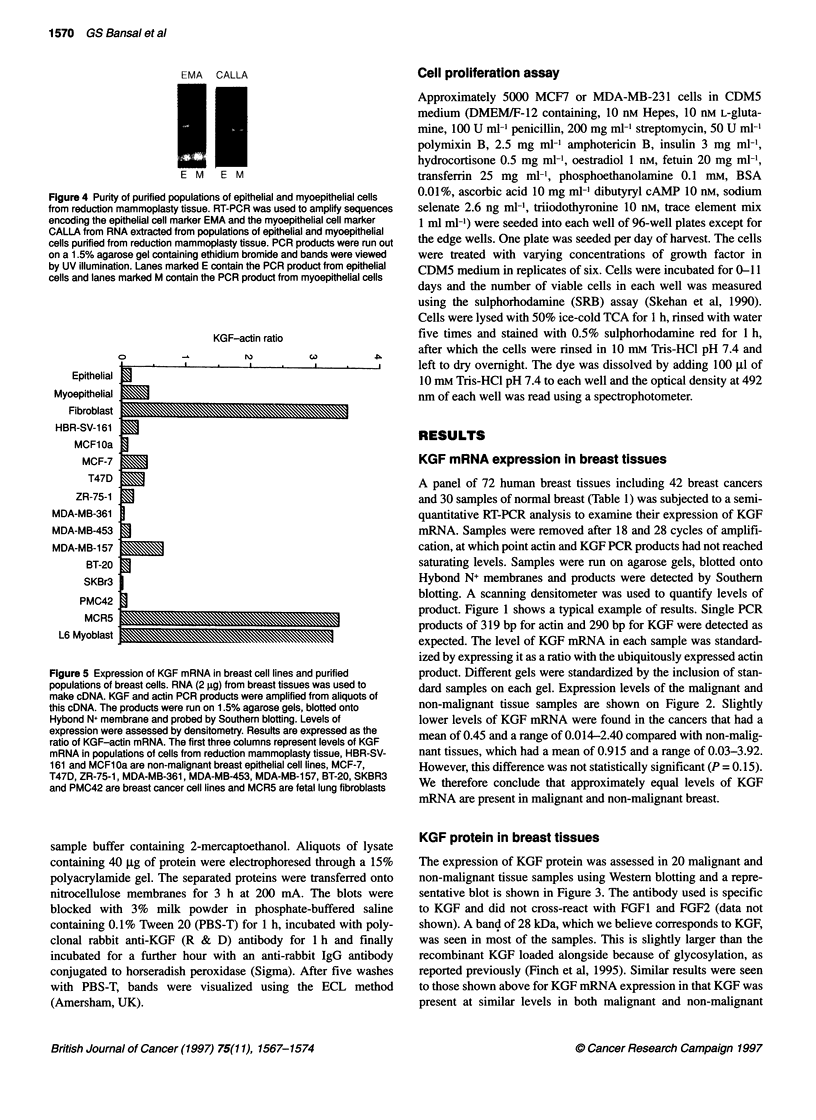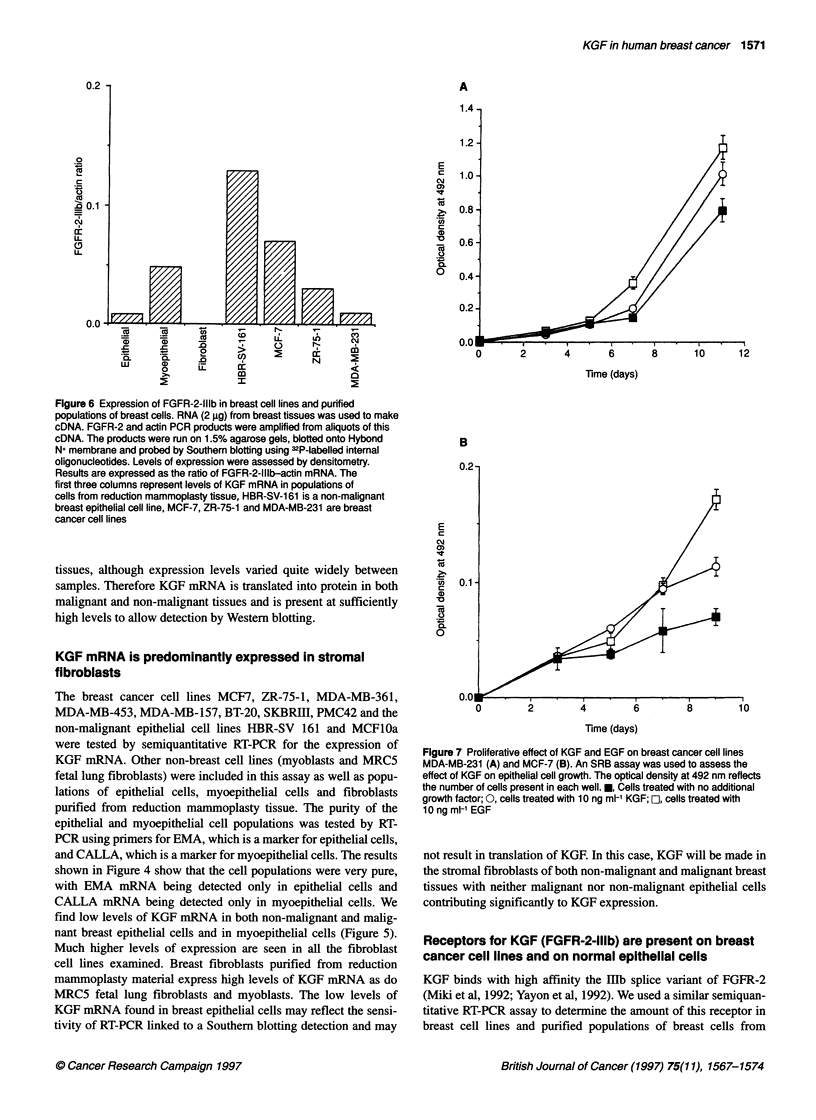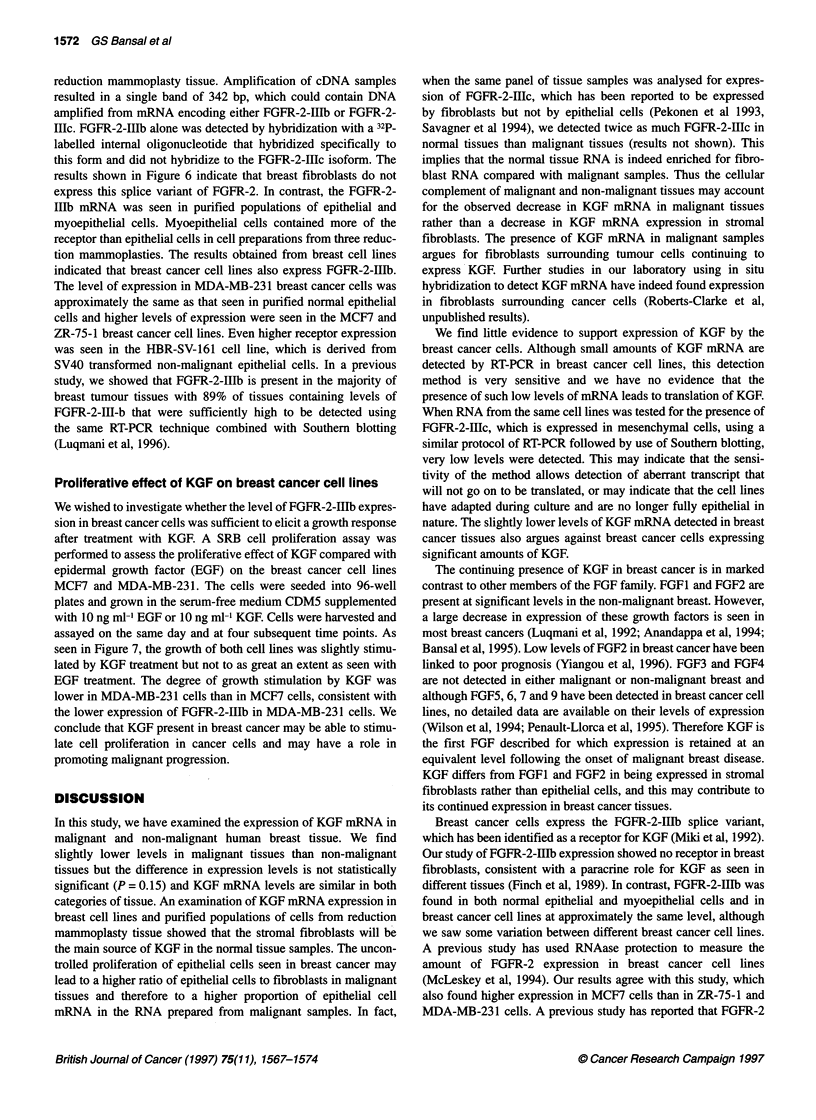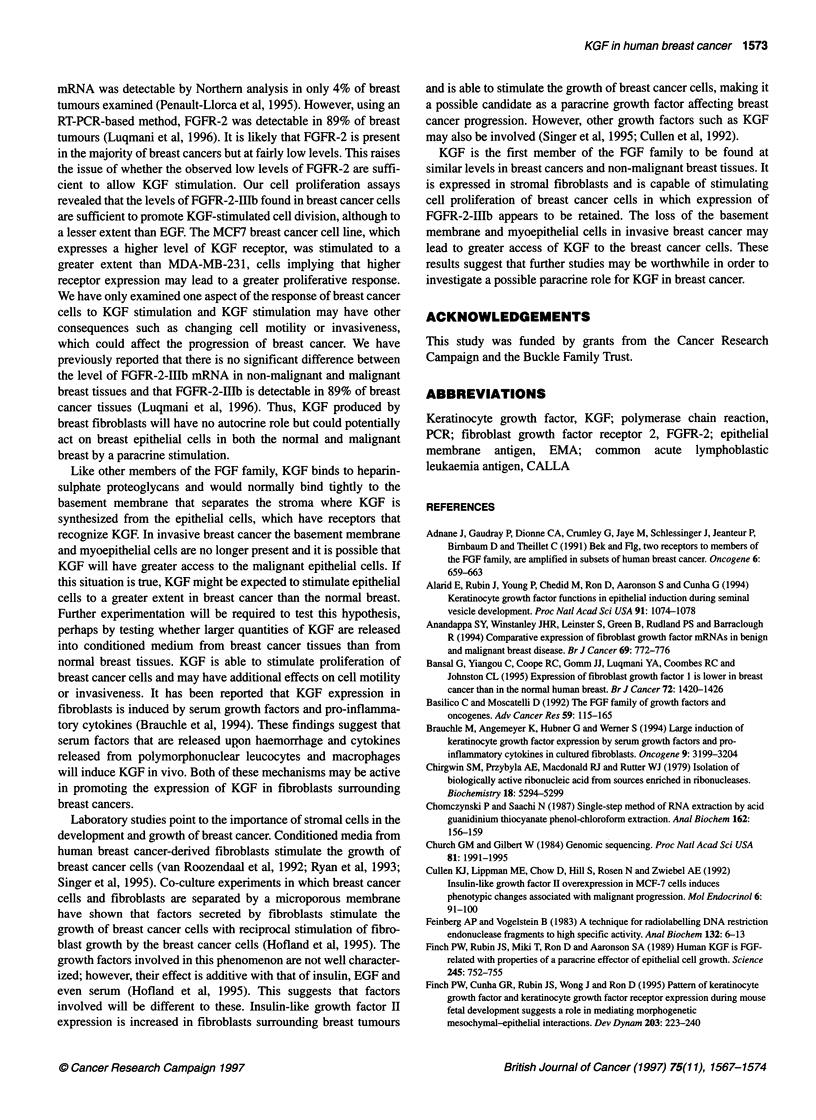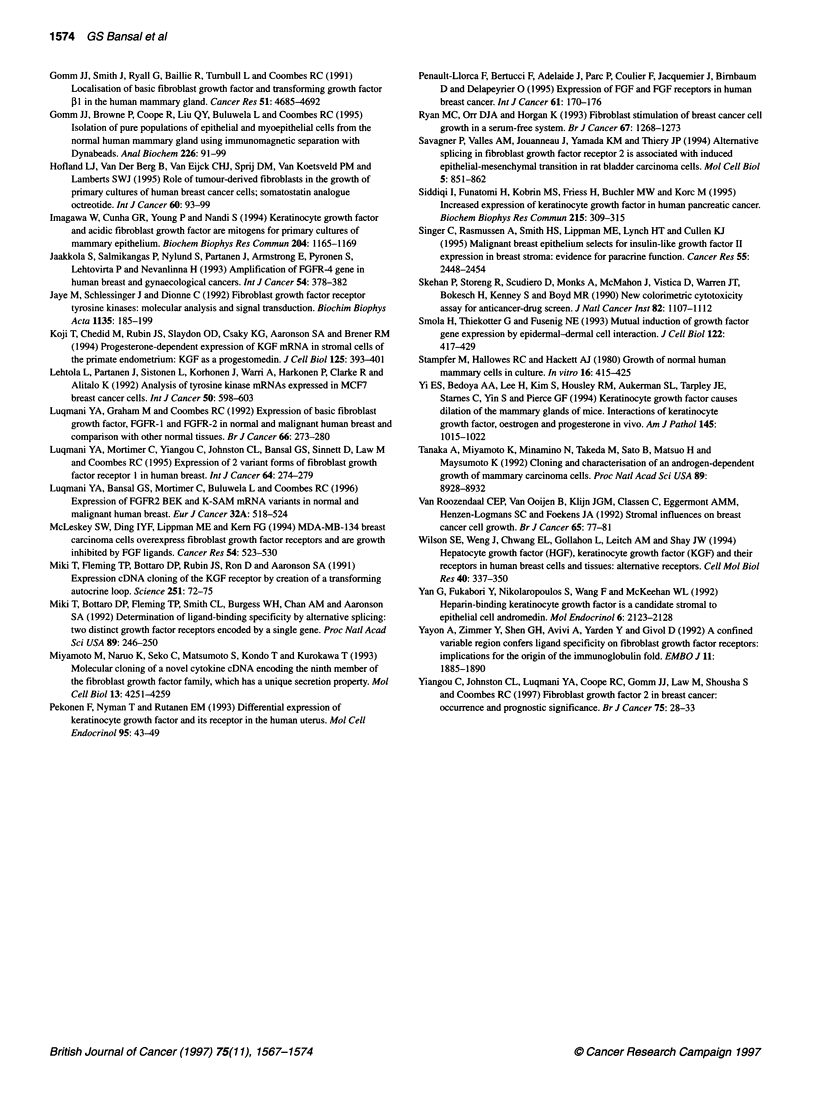# Expression of keratinocyte growth factor and its receptor in human breast cancer.

**DOI:** 10.1038/bjc.1997.269

**Published:** 1997

**Authors:** G. S. Bansal, H. C. Cox, S. Marsh, J. J. Gomm, C. Yiangou, Y. Luqmani, R. C. Coombes, C. L. Johnston

**Affiliations:** Department of Medical Oncology, Charing Cross and Westminster Medical School, London, UK.

## Abstract

**Images:**


British Journal of Cancer (1997) 75(11), 1567–1574
© 1997 Cancer Research Campaign

### Expression of keratinocyte growth factor and its receptor in human breast cancer

**GS Bansal, HC Cox, S Marsh, JJ Gomm, C Yiangou, Y Luqmani, RC Coombes and CL Johnston**

Department of Medical Oncology, Charing Cross and Westminster Medical School, St. Dunstans Road, London W6 8RF, UK

**Summary** The level of expression of keratinocyte growth factor (KGF) mRNA has been measured in human breast cell lines, purified populations of epithelial cells, myoepithelial cells and fibroblasts from reduction mammoplasty tissue and a panel of 42 breast cancers and 30 non-malignant human breast tissues using a semiquantitative reverse transcriptase polymerase chain reaction (RT-PCR) procedure. We found similar levels of KGF mRNA in malignant and non-malignant breast tissues. The study of the amount of KGF mRNA in breast cell lines and purified populations of cells revealed that fibroblasts are the predominant source of KGF with malignant and non-malignant epithelial cells containing very low levels of KGF mRNA. We have examined the distribution of fibroblast growth factor receptor (FGFR)-2-IIIb, which is a high-affinity receptor for KGF and find that it is present on malignant and non-malignant epithelial cells. The level of FGFR-2-IIIb present on breast cancer cell lines was sufficient for KGF stimulation of breast cancer cell proliferation. Other members of the fibroblast growth factor family have been either not expressed in the human breast (FGF3, FGF4) or have been found at much reduced levels in breast cancer (FGF1, FGF2) and this is the first member of the family to potentially influence the progression of breast cancer through stimulation of cell division.

**Keywords:** keratinocyte growth factor; FGF7; human breast cancer; fibroblast growth factor receptor 2

Fibroblast growth factors (FGFs) comprise a family of nine polypeptide mitogens including acidic FGF (FGF1), basic FGF (FGF2), int-2 (FGF3), hst (FGF4), FGF5, FGF6 and keratinocyte growth factor (KGF or FGF7) (Basilico and Moscatelli, 1992). Two more recently identified members of the family are androgen-induced growth factor (AIGF, FGF8) and glia-activating factor (GAF, FGF9) (Tanaka et al, 1992; Miyamoto et al, 1993). KGF differs from the other members by its high specificity for activating epithelial cells (Finch et al, 1989). It is expressed in cells of mesenchymal origin such as fibroblasts and endothelial cells but not in epithelial cells (Finch et al, 1989; Smola et al 1993). It therefore seems likely that KGF stimulates epithelial cells in a paracrine manner.

Four genes encoding high-affinity receptors for FGFs have been described and the complexity of this gene family is enhanced by extensive variation in splicing (Jaye et al, 1992). Each receptor has a similar structure of three extracellular immunoglobulin domains that are involved in ligand binding, a transmembrane domain and an intracellular split tyrosine kinase domain (Jaye et al, 1992). KGF binds to a splice variant of FGFR-2 (Miki et al, 1991). Alternative splicing of the carboxyl-terminal half of the third immunoglobulin-like domain changes the ligand-binding properties of FGFR-2 with FGFR-2-IIIb binding to FGF1 and KGF whereas FGFR-2-IIIc binds to FGF1 and FGF2 (Miki et al, 1992; Yayon et al, 1992). These two isoforms of FGFR-2 appear to be expressed in a mutually exclusive fashion in many cell systems. Cells of mesenchymal origin express FGFR-2-IIIc, whereas epithelial cells express FGFR-2-IIIb (Pekonen et al, 1993; Savagner et al, 1994).

*Received 11 September 1996*
*Revised 26 November 1996*
*Accepted 18 December 1996*

*Correspondence to:* Dr C Johnston

Several studies have examined the expression of FGFs in the malignant and non-malignant breast. mRNA encoding FGF1, FGF2, FGF5, FGF6, FGF7 and FGF9 has been detected in breast cancer, with expression of FGF1 and FGF2 found in all breast cancers whereas the others showed more restricted expression (Penault-Llorca et al, 1995). Quantitative studies comparing expression levels in malignant and non-malignant breast showed that FGF1 and FGF2 are present in considerable quantities in the non-malignant breast but their expression is greatly reduced in malignant tissues (Bansal et al, 1995; Anandappa et al, 1994; Luqmani et al, 1992). These results may indicate that FGF1 and FGF2 have a role in maintaining the normal ducts. FGF2 has been localized to the myoepithelial cells of the normal breast by immunocytochemistry but could not be detected in normal or malignant epithelial cells by this technique (Gomm et al, 1991). High-affinity receptors for FGFs are found in breast cancer cells. Amplification of the *FGFR-1* and *FGFR-2* genes was found in 12.7% and 11.5% of breast tumours respectively and gene amplification of *FGFR-4* was found in 10% of breast cancers (Adnane et al 1991, Jaakkola et al, 1993). Elevated levels of FGFR mRNAs were found in several breast cancer cell lines (Lehtola et al, 1992; McLeskey et al 1994). The expression of different splice variants of FGF receptors could also be important in breast cancer and a high ratio of beta to alpha form expression of FGFR-1 has been associated with a reduced disease-free survival in patients with breast cancer (Luqmani et al 1995).

In this study, we have addressed the issue of whether KGF has a role in the human breast and the development of breast cancer. KGF is an androgen-induced stromal growth factor that can stimulate epithelial growth and morphogenesis in the developing prostate and seminal vesicle (Yan et al, 1992; Alarid et al, 1994). KGF has also been shown to be a progestomedin in the endometrium of primates (Koji et al, 1994). Its role in the mammary gland, another steroid hormone-dependent tissue, is less

Table 1 Details of patients studied

| Characteristic | Number of patients | Patients (%) |
|---|---|---|
| Total number of patients | 42 | |
| Age range | 29–76 | |
| Mean age | 65 | |
| Menopausal status | | |
| Pre | 11 | 30 |
| Post | 26 | 70 |
| Not known | 5 | |
| Clinical stage | | |
| T1/T2 | 28 | 90 |
| T3/T4 | 4 | 10 |
| Not known | 10 | |
| Node status | | |
| Negative | 18 | 60 |
| Positive | 12 | 40 |
| Not known | 12 | |
| ER status | | |
| Positive | 9 | 60 |
| Negative | 6 | 40 |
| Not known | 27 | |
| Histological type | | |
| Infiltrating ductal | 33 | 89 |
| Infiltrating lobular | 4 | 11 |
| Not known | 5 | |

well defined. However, studies in mice have shown that KGF is a mitogen for primary cultures of mammary epithelium (Imagawa et al, 1994) and systemic administration of KGF leads to hyperproliferation of the mammary gland, giving the histological appearance of fibrocystic disease (Yi et al, 1994). KGF has been linked to human pancreatic cancer with 44% of pancreatic cancer samples showing overexpression of KGF mRNA by Northern analysis (Siddiqi et al, 1995). Our results indicate that KGF mRNA is present at approximately the same levels in non-malignant and malignant breast tissue, where it is expressed by stromal cells and is able to stimulate epithelial cells. It may have a role in normal mammary development and morphogenesis and, in contrast to FGF1 and FGF2, is retained in malignant tissue where it could potentially influence the growth of breast cancers.

### MATERIALS AND METHODS

Reverse transcriptase was from Gibco-BRL (Paisley, UK), *Taq* polymerase from Peninsula Laboratories (UK), DNA polymerase klenow fragment and dNTPs from Pharmacia (Uppsala, Sweden). RNAzol was from Biogenesis (Bournemouth, UK). [$\alpha$-$^{32}$P]dCTP (3000 Ci mmol$^{-1}$) and Hybond N$^{+}$ membranes and Hyperfilm were from Amersham (UK). All other reagents were obtained from Sigma (Poole, UK) unless otherwise indicated and were of the highest available grade.

### Cell lines

Twelve human mammary cell lines were used in this study: two breast cell lines of non-malignant origin, HBR-SV1.6.1 (epithelial) and MCF10a (epithelial), and ten derived from cancer tissue; T47D, ZR-75-1, SKBR111, MDA-MB-231, MDA-MB-361, MDA-MB-453, MDA-MB-157, BT20, PMC42 and MCF7. A further two non-breast cell lines rat L6 myoblasts and MCR5 fetal lung fibroblasts were also analysed for comparison. All but three of these cell lines were cultured in RPMI-1640 medium buffered with 25 mM Hepes and supplemented with 10% fetal calf serum (FCS), 100 units ml$^{-1}$ penicillin, 100 μg ml$^{-1}$ streptomycin and 2 mM L-glutamine. The SKBR111 cells were grown in McCoy's 5A medium with the same supplements as above and the MCF10a cells in a medium containing equal quantities of Dulbecco's modified eagle medium (DMEM) and Ham's nutrient mixture F-12 buffered with 15 mM Hepes with the following supplements: 10 μg ml$^{-1}$ insulin, 1.4 nM hydrocortisone, 100 ng ml$^{-1}$ cholera enterotoxin, 20 ng ml$^{-1}$ epidermal growth factor, 5% horse serum, 2 mM glutamine, 100 units ml$^{-1}$ penicillin and 100 μg ml$^{-1}$ streptomycin. Primary cultures of breast fibroblasts were grown in DMEM: Ham's F12 1:1 with 15 mM Hepes and 100 U ml$^{-1}$ penicillin, 100 μg ml$^{-1}$ streptomycin, 5 μg ml$^{-1}$ amphotericin B, 50 U ml$^{-1}$ polymixin B, 10 μg ml$^{-1}$ insulin, 10 ng ml$^{-1}$ EGF, 1 μg ml$^{-1}$ hydrocortisone, 10 μg ml$^{-1}$ transferrin, 0.1 mM ethanolamine and 5% FCS. Cells were harvested at about 80% confluence.

### Preparation of pure populations of breast epithelial and myoepithelial cells

Separated breast fibroblasts, epithelial and myoepithelial cells were prepared from reduction mammoplasty specimens by immunomagnetic separation using the method of Gomm et al (1995). Mouse monoclonal antibodies to common acute lymphoblastic leukaemia antigen (CALLA) (Sera-lab) and epithelial membrane antigen (EMA) (Sera-lab) were bound to anti-mouse and anti-rat antibody-coated beads respectively (Dynabeads) overnight at 4°C and then washed four times with cold medium. Breast organoids were prepared from reduction mammoplasty tissue by a modification of the method of Stampfer et al (1980). Single cell suspensions were prepared by digestion of organoids in PBS containing trypsin–EDTA (0.05%/0.02%) and 0.4 mg ml$^{-1}$ DNAase for 15 min at 37°C. Cells were washed three times in medium and filtered through 56-μm gauze. The cells were incubated with anti-CALLA- or anti-EMA-coated beads in a ratio of ten beads per target cell. Three separate incubations of 15 min at 4°C were performed to collect cells.

### RT–PCR analysis of purified populations of breast cells to assess their purity

Cellular RNA was extracted from the purified fractions of cells by the modified RNAzol procedure (Chomczynsky and Saatchi, 1987). RNA (2 μg) was reverse transcribed using random primers. cDNA was amplified using 1 unit of *Taq* polymerase in 100 μl containing 67 mM Tris-HCl pH 8.8, 1.5 mM magnesium chloride, 16 mM ammonium sulphate, 0.45% Triton X-100, 200 μg ml$^{-1}$ gelatin, 200 μM$^{-1}$ dNTPs and 200 ng of each of the EMA or CALLA primers, by 40 sequential cycles of denaturation at 95°C for 1 min, annealing at 55°C for 1 min and extension at 72°C for 1 min (extended to 10 min for the final cycle). Aliquots (10 μl) of the 40 cycle PCR products were electrophoresed on a 1% agarose gel containing ethidium bromide and bands were visualized by UV illumination.

© Cancer Research Campaign 1997

**Figure 1** Southern blot of RT-PCR products for KGF and actin amplified from malignant and non-malignant tissues. RNA (2 μg) from breast tissues was used to make cDNA. KGF and actin PCR products were amplified from aliquots of this cDNA. The products were run on 1.5% agarose gels, blotted onto Hybond N+ membrane and $^{32}$P-labelled probes were used to identify and quantify bands. Lanes 1–9 contain PCR products from breast cancer tissues, lanes 10–11 contain PCR products from non-malignant breast tissues

**Figure 2** Expression of KGF mRNA in malignant and non-malignant human breast tissues. Levels of expression were assessed from Southern blots by densitometry. Results are expressed as the ratio of KGF–actin mRNA. ◇, Levels of KGF mRNA in non-malignant tissues; ◆, levels of KGF expression in breast cancers

**Figure 3** Expression of KGF protein in malignant and non-malignant human breast tissues. Tissue lysates of cancer (C) and non-malignant (N) tissues and a recombinant KGF protein were run on a 15% acrylamide gel and blotted onto nitrocellulose. The blot was probed with a rabbit polyclonal antibody against KGF and anti-rabbit horseradish peroxidase and bands were visualized using ECL reagents

### Tissues

Breast tissue obtained at surgery was snap frozen and stored in liquid nitrogen. We collected cancer tissue from 42 patients whose details are given in Table 1, showing these to be a typically representative cohort of breast cancer patients with 30% of patients being pre/perimenopausal and 40% having oestrogen receptor-positive carcinomas. Breast tissue adjacent to carcinoma or from benign conditions histologically confirmed to be non-malignant was also collected and is referred to as normal.

### Oligonucleotides

Oligonucleotide primers were synthesized on a Cyclone Plus DNA Synthesizer (Milligan Bioresearch, MA, USA). The primers used for the PCR were: for KGF, 5′-ATGGAAATCAGGACAGTGGC-3′ (sense) and 5′-CATAGGAAGAAAGTGGCCTG-3′ (antisense); for FGFR-2,5′-CTGGATGTTGTGGAGCGAT-3′ (sense) and 5′-TGTAATCTCCTTTTCTCTT CCA-3′ (antisense); for actin, 5′-CATCTCTTGCTCGAAGAAGTCCA-3′ and 5′-ATCATGTTT-GAGACCTTCAA-3′; for EMA, 5′-TCCGCTCCACCTCT-CAAG-3′ (sense) and 5′-CTCACAGCATTCTTCTCAGTAG-3′ (antisense); and for CALLA, 5′-TTGTAAGCAGCCTCAGCC-3′ (sense) and 5′-TTGTCCACCTTTTCTCGG-3′ (antisense). The FGFR-2-IIIb PCR product was detected specifically by hybridizing with the $^{32}$P-labelled internal oligonucleotide 5′-TGGGAACTATTTATCCCCG-3′ (antisense).

### Determination of KGF and FGFR-2-IIIb mRNA by RT-PCR amplification

Cellular RNA was extracted from pulverized frozen tissues using the guanidiniumisothiocyanate method (Chirgwin et al, 1979) and from the cell lines by the modified RNAzol procedure (Chomczynsky and Saatchi, 1987). Reverse transcription and PCR amplification was performed as described previously (Luqmani et al, 1992). Briefly, 2 μg of RNA was reverse transcribed using random primers and cDNA was amplified using 1 unit *Taq* polymerase in 100 μl containing 67 mM Tris-HCl pH 8.8, 1.5 mM magnesium chloride, 16 mM ammonium sulphate, 0.45% Triton X-100, 200 μg $ml^{-1}$ gelatin, 200 μM dNTPs and 200 ng of each of the KGF or FGFR-2 and actin primers, by sequential cycles of denaturation at 95°C for 1 min, annealing at 55°C (or 45°C for FGFR-2) for 1 min and extension at 72°C for 1 min (extended to 10 min for the final cycle). An aliquot was removed after 18 cycles for estimation of actin product and the reaction continued for a further ten cycles for estimation of KGF and FGFR-2. Aliquots (10 μl) of the 28 cycle and 18 cycle PCR products were electrophoresed on separate 1% agarose gels and alkali blotted overnight onto Hybond N+ membrane (Luqmani et al 1992).

Hybridization was carried out as described by Church and Gilbert (1984). We initially used plasmids containing KGF or actin cDNA for hybridizations to verify identity and size of PCR products. As single bands were seen, we subsequently used PCR products (made using plasmid template) random primer labelled (Feinberg and Vogelstein, 1983) with [$^{32}$P]dCTP ($5 \times 10^8$ to $5 \times 10^9$ c.p.m. $\mu g^{-1}$, $5 \times 10^6$ c.p.m. $ml^{-1}$). In the case of FGFR-2, the $^{32}$P-labelled internal oligonucleotide 5′-TGGGAACTATTTATC-CCCG-3′ (antisense), which hybridizes to FGFR-2-IIIb but not to FGFR-2-IIIc, was used to detect FGFR-2-IIIb. Washed blots were exposed to photographic film for several hours and band intensities were quantified by densitometry. The values for KGF and FGFR-2-IIIb were normalized by dividing the signal for KGF or FGFR-2-IIIb by that for actin. Separate blots were normalized to each other by using an arbitary sample that was present on every run and every blot to correct for differences between experiments.

### Detection of KGF by western blotting

The tissues were lysed in PBS containing 1% NP40, 0.1% SDS, 100 μg $ml^{-1}$ phenylmethylsulphonyl fluoride (PMSF) and 5 μg $ml^{-1}$ aprotinin and then mixed with an equal volume of SDS–PAGE

© Cancer Research Campaign 1997

**Figure 4** Purity of purified populations of epithelial and myoepithelial cells from reduction mammoplasty tissue. RT-PCR was used to amplify sequences encoding the epithelial cell marker EMA and the myoepithelial cell marker CALLA from RNA extracted from populations of epithelial and myoepithelial cells purified from reduction mammoplasty tissue. PCR products were run out on a 1.5% agarose gel containing ethidium bromide and bands were viewed by UV illumination. Lanes marked E contain the PCR product from epithelial cells and lanes marked M contain the PCR product from myoepithelial cells

**Figure 5** Expression of KGF mRNA in breast cell lines and purified populations of breast cells. RNA (2 μg) from breast tissues was used to make cDNA. KGF and actin PCR products were amplified from aliquots of this cDNA. The products were run on 1.5% agarose gels, blotted onto Hybond N+ membrane and probed by Southern blotting. Levels of expression were assessed by densitometry. Results are expressed as the ratio of KGF–actin mRNA. The first three columns represent levels of KGF mRNA in populations of cells from reduction mammoplasty tissue, HBR-SV-161 and MCF10a are non-malignant breast epithelial cell lines, MCF-7, T47D, ZR-75-1, MDA-MB-361, MDA-MB-453, MDA-MB-157, BT-20, SKBR3 and PMC42 are breast cancer cell lines and MCR5 are fetal lung fibroblasts sample buffer containing 2-mercaptoethanol. Aliquots of lysate containing 40 μg of protein were electrophoresed through a 15% polyacrylamide gel. The separated proteins were transferred onto nitrocellulose membranes for 3 h at 200 mA. The blots were blocked with 3% milk powder in phosphate-buffered saline containing 0.1% Tween 20 (PBS-T) for 1 h, incubated with polyclonal rabbit anti-KGF (R & D) antibody for 1 h and finally incubated for a further hour with an anti-rabbit IgG antibody conjugated to horseradish peroxidase (Sigma). After five washes with PBS-T, bands were visualized using the ECL method (Amersham, UK).

### Cell proliferation assay

Approximately 5000 MCF7 or MDA-MB-231 cells in CDM5 medium (DMEM/F-12 containing, 10 nM Hepes, 10 nM L-glutamine, 100 U $ml^{-1}$ penicillin, 200 mg $ml^{-1}$ streptomycin, 50 U $ml^{-1}$ polymixin B, 2.5 mg $ml^{-1}$ amphotericin B, insulin 3 mg $ml^{-1}$, hydrocortisone 0.5 mg $ml^{-1}$, oestradiol 1 nM, fetuin 20 mg $ml^{-1}$, transferrin 25 mg $ml^{-1}$, phosphoethanolamine 0.1 mM, BSA 0.01%, ascorbic acid 10 mg $ml^{-1}$ dibutyryl cAMP 10 nM, sodium selenate 2.6 ng $ml^{-1}$, triiodothyronine 10 nM, trace element mix 1 ml $ml^{-1}$) were seeded into each well of 96-well plates except for the edge wells. One plate was seeded per day of harvest. The cells were treated with varying concentrations of growth factor in CDM5 medium in replicates of six. Cells were incubated for 0–11 days and the number of viable cells in each well was measured using the sulphorhodamine (SRB) assay (Skehan et al, 1990). Cells were lysed with 50% ice-cold TCA for 1 h, rinsed with water five times and stained with 0.5% sulphorhodamine red for 1 h, after which the cells were rinsed in 10 mM Tris-HCl pH 7.4 and left to dry overnight. The dye was dissolved by adding 100 μl of 10 mM Tris-HCl pH 7.4 to each well and the optical density at 492 nm of each well was read using a spectrophotometer.

### RESULTS

### KGF mRNA expression in breast tissues

A panel of 72 human breast tissues including 42 breast cancers and 30 samples of normal breast (Table 1) was subjected to a semi-quantitative RT-PCR analysis to examine their expression of KGF mRNA. Samples were removed after 18 and 28 cycles of amplification, at which point actin and KGF PCR products had not reached saturating levels. Samples were run on agarose gels, blotted onto Hybond N+ membranes and products were detected by Southern blotting. A scanning densitometer was used to quantify levels of product. Figure 1 shows a typical example of results. Single PCR products of 319 bp for actin and 290 bp for KGF were detected as expected. The level of KGF mRNA in each sample was standardized by expressing it as a ratio with the ubiquitously expressed actin product. Different gels were standardized by the inclusion of standard samples on each gel. Expression levels of the malignant and non-malignant tissue samples are shown on Figure 2. Slightly lower levels of KGF mRNA were found in the cancers that had a mean of 0.45 and a range of 0.014–2.40 compared with non-malignant tissues, which had a mean of 0.915 and a range of 0.03–3.92. However, this difference was not statistically significant ($P = 0.15$). We therefore conclude that approximately equal levels of KGF mRNA are present in malignant and non-malignant breast.

### KGF protein in breast tissues

The expression of KGF protein was assessed in 20 malignant and non-malignant tissue samples using Western blotting and a representative blot is shown in Figure 3. The antibody used is specific to KGF and did not cross-react with FGF1 and FGF2 (data not shown). A band of 28 kDa, which we believe corresponds to KGF, was seen in most of the samples. This is slightly larger than the recombinant KGF loaded alongside because of glycosylation, as reported previously (Finch et al, 1995). Similar results were seen to those shown above for KGF mRNA expression in that KGF was present at similar levels in both malignant and non-malignant

 © Cancer Research Campaign 1997

**Figure 6** Expression of FGFR-2-IIIb in breast cell lines and purified populations of breast cells. RNA (2 μg) from breast tissues was used to make cDNA. FGFR-2 and actin PCR products were amplified from aliquots of this cDNA. The products were run on 1.5% agarose gels, blotted onto Hybond N+ membrane and probed by Southern blotting using $^{32}P$-labelled internal oligonucleotides. Levels of expression were assessed by densitometry. Results are expressed as the ratio of FGFR-2-IIIb–actin mRNA. The first three columns represent levels of KGF mRNA in populations of cells from reduction mammoplasty tissue, HBR-SV-161 is a non-malignant breast epithelial cell line, MCF-7, ZR-75-1 and MDA-MB-231 are breast cancer cell lines

**Figure 7** Proliferative effect of KGF and EGF on breast cancer cell lines MDA-MB-231 (**A**) and MCF-7 (**B**). An SRB assay was used to assess the effect of KGF on epithelial cell growth. The optical density at 492 nm reflects the number of cells present in each well. ■, Cells treated with no additional growth factor; ○, cells treated with 10 ng ml$^{-1}$ KGF; □, cells treated with 10 ng ml$^{-1}$ EGF

tissues, although expression levels varied quite widely between samples. Therefore KGF mRNA is translated into protein in both malignant and non-malignant tissues and is present at sufficiently high levels to allow detection by Western blotting.

### KGF mRNA is predominantly expressed in stromal fibroblasts

The breast cancer cell lines MCF7, ZR-75-1, MDA-MB-361, MDA-MB-453, MDA-MB-157, BT-20, SKBRIII, PMC42 and the non-malignant epithelial cell lines HBR-SV 161 and MCF10a were tested by semiquantitative RT-PCR for the expression of KGF mRNA. Other non-breast cell lines (myoblasts and MRC5 fetal lung fibroblasts) were included in this assay as well as populations of epithelial cells, myoepithelial cells and fibroblasts purified from reduction mammoplasty tissue. The purity of the epithelial and myoepithelial cell populations was tested by RT-PCR using primers for EMA, which is a marker for epithelial cells, and CALLA, which is a marker for myoepithelial cells. The results shown in Figure 4 show that the cell populations were very pure, with EMA mRNA being detected only in epithelial cells and CALLA mRNA being detected only in myoepithelial cells. We find low levels of KGF mRNA in both non-malignant and malignant breast epithelial cells and in myoepithelial cells (Figure 5). Much higher levels of expression are seen in all the fibroblast cell lines examined. Breast fibroblasts purified from reduction mammoplasty material express high levels of KGF mRNA as do MRC5 fetal lung fibroblasts and myoblasts. The low levels of KGF mRNA found in breast epithelial cells may reflect the sensitivity of RT-PCR linked to a Southern blotting detection and may not result in translation of KGF. In this case, KGF will be made in the stromal fibroblasts of both non-malignant and malignant breast tissues with neither malignant nor non-malignant epithelial cells contributing significantly to KGF expression.

### Receptors for KGF (FGFR-2-IIIb) are present on breast cancer cell lines and on normal epithelial cells

KGF binds with high affinity the IIIb splice variant of FGFR-2 (Miki et al, 1992; Yayon et al, 1992). We used a similar semiquantitative RT-PCR assay to determine the amount of this receptor in breast cell lines and purified populations of breast cells from

*© Cancer Research Campaign 1997*

reduction mammoplasty tissue. Amplification of cDNA samples resulted in a single band of 342 bp, which could contain DNA amplified from mRNA encoding either FGFR-2-IIIb or FGFR-2-IIIc. FGFR-2-IIIb alone was detected by hybridization with a $^{32}$P-labelled internal oligonucleotide that hybridized specifically to this form and did not hybridize to the FGFR-2-IIIc isoform. The results shown in Figure 6 indicate that breast fibroblasts do not express this splice variant of FGFR-2. In contrast, the FGFR-2-IIIb mRNA was seen in purified populations of epithelial and myoepithelial cells. Myoepithelial cells contained more of the receptor than epithelial cells in cell preparations from three reduction mammoplasties. The results obtained from breast cell lines indicated that breast cancer cell lines also express FGFR-2-IIIb. The level of expression in MDA-MB-231 breast cancer cells was approximately the same as that seen in purified normal epithelial cells and higher levels of expression were seen in the MCF7 and ZR-75-1 breast cancer cell lines. Even higher receptor expression was seen in the HBR-SV-161 cell line, which is derived from SV40 transformed non-malignant epithelial cells. In a previous study, we showed that FGFR-2-IIIb is present in the majority of breast tumour tissues with 89% of tissues containing levels of FGFR-2-III-b that were sufficiently high to be detected using the same RT-PCR technique combined with Southern blotting (Luqmani et al, 1996).

### Proliferative effect of KGF on breast cancer cell lines

We wished to investigate whether the level of FGFR-2-IIIb expression in breast cancer cells was sufficient to elicit a growth response after treatment with KGF. A SRB cell proliferation assay was performed to assess the proliferative effect of KGF compared with epidermal growth factor (EGF) on the breast cancer cell lines MCF7 and MDA-MB-231. The cells were seeded into 96-well plates and grown in the serum-free medium CDM5 supplemented with 10 ng ml$^{-1}$ EGF or 10 ng ml$^{-1}$ KGF. Cells were harvested and assayed on the same day and at four subsequent time points. As seen in Figure 7, the growth of both cell lines was slightly stimulated by KGF treatment but not to as great an extent as seen with EGF treatment. The degree of growth stimulation by KGF was lower in MDA-MB-231 cells than in MCF7 cells, consistent with the lower expression of FGFR-2-IIIb in MDA-MB-231 cells. We conclude that KGF present in breast cancer may be able to stimulate cell proliferation in cancer cells and may have a role in promoting malignant progression.

### DISCUSSION

In this study, we have examined the expression of KGF mRNA in malignant and non-malignant human breast tissue. We find slightly lower levels in malignant tissues than non-malignant tissues but the difference in expression levels is not statistically significant ($P = 0.15$) and KGF mRNA levels are similar in both categories of tissue. An examination of KGF mRNA expression in breast cell lines and purified populations of cells from reduction mammoplasty tissue showed that the stromal fibroblasts will be the main source of KGF in the normal tissue samples. The uncontrolled proliferation of epithelial cells seen in breast cancer may lead to a higher ratio of epithelial cells to fibroblasts in malignant tissues and therefore to a higher proportion of epithelial cell mRNA in the RNA prepared from malignant samples. In fact, when the same panel of tissue samples was analysed for expression of FGFR-2-IIIc, which has been reported to be expressed by fibroblasts but not by epithelial cells (Pekonen et al 1993, Savagner et al 1994), we detected twice as much FGFR-2-IIIc in normal tissues than malignant tissues (results not shown). This implies that the normal tissue RNA is indeed enriched for fibroblast RNA compared with malignant samples. Thus the cellular complement of malignant and non-malignant tissues may account for the observed decrease in KGF mRNA in malignant tissues rather than a decrease in KGF mRNA expression in stromal fibroblasts. The presence of KGF mRNA in malignant samples argues for fibroblasts surrounding tumour cells continuing to express KGF. Further studies in our laboratory using in situ hybridization to detect KGF mRNA have indeed found expression in fibroblasts surrounding cancer cells (Roberts-Clarke et al, unpublished results).

We find little evidence to support expression of KGF by the breast cancer cells. Although small amounts of KGF mRNA are detected by RT-PCR in breast cancer cell lines, this detection method is very sensitive and we have no evidence that the presence of such low levels of mRNA leads to translation of KGF. When RNA from the same cell lines was tested for the presence of FGFR-2-IIIc, which is expressed in mesenchymal cells, using a similar protocol of RT-PCR followed by use of Southern blotting, very low levels were detected. This may indicate that the sensitivity of the method allows detection of aberrant transcript that will not go on to be translated, or may indicate that the cell lines have adapted during culture and are no longer fully epithelial in nature. The slightly lower levels of KGF mRNA detected in breast cancer tissues also argues against breast cancer cells expressing significant amounts of KGF.

The continuing presence of KGF in breast cancer is in marked contrast to other members of the FGF family. FGF1 and FGF2 are present at significant levels in the non-malignant breast. However, a large decrease in expression of these growth factors is seen in most breast cancers (Luqmani et al, 1992; Anandappa et al, 1994; Bansal et al, 1995). Low levels of FGF2 in breast cancer have been linked to poor prognosis (Yiangou et al, 1996). FGF3 and FGF4 are not detected in either malignant or non-malignant breast and although FGF5, 6, 7 and 9 have been detected in breast cancer cell lines, no detailed data are available on their levels of expression (Wilson et al, 1994; Penault-Llorca et al, 1995). Therefore KGF is the first FGF described for which expression is retained at an equivalent level following the onset of malignant breast disease. KGF differs from FGF1 and FGF2 in being expressed in stromal fibroblasts rather than epithelial cells, and this may contribute to its continued expression in breast cancer tissues.

Breast cancer cells express the FGFR-2-IIIb splice variant, which has been identified as a receptor for KGF (Miki et al, 1992). Our study of FGFR-2-IIIb expression showed no receptor in breast fibroblasts, consistent with a paracrine role for KGF as seen in different tissues (Finch et al, 1989). In contrast, FGFR-2-IIIb was found in both normal epithelial and myoepithelial cells and in breast cancer cell lines at approximately the same level, although we saw some variation between different breast cancer cell lines. A previous study has used RNAase protection to measure the amount of FGFR-2 expression in breast cancer cell lines (McLeskey et al, 1994). Our results agree with this study, which also found higher expression in MCF7 cells than in ZR-75-1 and MDA-MB-231 cells. A previous study has reported that FGFR-2

 © Cancer Research Campaign 1997

mRNA was detectable by Northern analysis in only 4% of breast tumours examined (Penault-Llorca et al, 1995). However, using an RT-PCR-based method, FGFR-2 was detectable in 89% of breast tumours (Luqmani et al, 1996). It is likely that FGFR-2 is present in the majority of breast cancers but at fairly low levels. This raises the issue of whether the observed low levels of FGFR-2 are sufficient to allow KGF stimulation. Our cell proliferation assays revealed that the levels of FGFR-2-IIIb found in breast cancer cells are sufficient to promote KGF-stimulated cell division, although to a lesser extent than EGF. The MCF7 breast cancer cell line, which expresses a higher level of KGF receptor, was stimulated to a greater extent than MDA-MB-231, cells implying that higher receptor expression may lead to a greater proliferative response. We have only examined one aspect of the response of breast cancer cells to KGF stimulation and KGF stimulation may have other consequences such as changing cell motility or invasiveness, which could affect the progression of breast cancer. We have previously reported that there is no significant difference between the level of FGFR-2-IIIb mRNA in non-malignant and malignant breast tissues and that FGFR-2-IIIb is detectable in 89% of breast cancer tissues (Luqmani et al, 1996). Thus, KGF produced by breast fibroblasts will have no autocrine role but could potentially act on breast epithelial cells in both the normal and malignant breast by a paracrine stimulation.

Like other members of the FGF family, KGF binds to heparin-sulphate proteoglycans and would normally bind tightly to the basement membrane that separates the stroma where KGF is synthesized from the epithelial cells, which have receptors that recognize KGF. In invasive breast cancer the basement membrane and myoepithelial cells are no longer present and it is possible that KGF will have greater access to the malignant epithelial cells. If this situation is true, KGF might be expected to stimulate epithelial cells to a greater extent in breast cancer than the normal breast. Further experimentation will be required to test this hypothesis, perhaps by testing whether larger quantities of KGF are released into conditioned medium from breast cancer tissues than from normal breast tissues. KGF is able to stimulate proliferation of breast cancer cells and may have additional effects on cell motility or invasiveness. It has been reported that KGF expression in fibroblasts is induced by serum growth factors and pro-inflammatory cytokines (Brauchle et al, 1994). These findings suggest that serum factors that are released upon haemorrhage and cytokines released from polymorphonuclear leucocytes and macrophages will induce KGF in vivo. Both of these mechanisms may be active in promoting the expression of KGF in fibroblasts surrounding breast cancers.

Laboratory studies point to the importance of stromal cells in the development and growth of breast cancer. Conditioned media from human breast cancer-derived fibroblasts stimulate the growth of breast cancer cells (van Roozendaal et al, 1992; Ryan et al, 1993; Singer et al, 1995). Co-culture experiments in which breast cancer cells and fibroblasts are separated by a microporous membrane have shown that factors secreted by fibroblasts stimulate the growth of breast cancer cells with reciprocal stimulation of fibroblast growth by the breast cancer cells (Hofland et al, 1995). The growth factors involved in this phenomenon are not well characterized; however, their effect is additive with that of insulin, EGF and even serum (Hofland et al, 1995). This suggests that factors involved will be different to these. Insulin-like growth factor II expression is increased in fibroblasts surrounding breast tumours and is able to stimulate the growth of breast cancer cells, making it a possible candidate as a paracrine growth factor affecting breast cancer progression. However, other growth factors such as KGF may also be involved (Singer et al, 1995; Cullen et al, 1992).

KGF is the first member of the FGF family to be found at similar levels in breast cancers and non-malignant breast tissues. It is expressed in stromal fibroblasts and is capable of stimulating cell proliferation of breast cancer cells in which expression of FGFR-2-IIIb appears to be retained. The loss of the basement membrane and myoepithelial cells in invasive breast cancer may lead to greater access of KGF to the breast cancer cells. These results suggest that further studies may be worthwhile in order to investigate a possible paracrine role for KGF in breast cancer.

### ACKNOWLEDGEMENTS

This study was funded by grants from the Cancer Research Campaign and the Buckle Family Trust.

### ABBREVIATIONS

Keratinocyte growth factor, KGF; polymerase chain reaction, PCR; fibroblast growth factor receptor 2, FGFR-2; epithelial membrane antigen, EMA; common acute lymphoblastic leukaemia antigen, CALLA

### REFERENCES

Adnane J, Gaudray P, Dionne CA, Crumley G, Jaye M, Schlessinger J, Jeanteur P, Birnbaum D and Theillet C (1991) Bek and Flg, two receptors to members of the FGF family, are amplified in subsets of human breast cancer. *Oncogene* **6**: 659–663

Alarid E, Rubin J, Young P, Chedid M, Ron D, Aaronson S and Cunha G (1994) Keratinocyte growth factor functions in epithelial induction during seminal vesicle development. *Proc Natl Acad Sci USA* **91**: 1074–1078

Anandappa SY, Winstanley JHR, Leinster S, Green B, Rudland PS and Barraclough R (1994) Comparative expression of fibroblast growth factor mRNAs in benign and malignant breast disease. *Br J Cancer* **69**: 772–776

Bansal G, Yiangou C, Coope RC, Gomm JJ, Luqmani YA, Coombes RC and Johnston CL (1995) Expression of fibroblast growth factor 1 is lower in breast cancer than in the normal human breast. *Br J Cancer* **72**: 1420–1426

Basilico C and Moscatelli D (1992) The FGF family of growth factors and oncogenes. *Adv Cancer Res* **59**: 115–165

Brauchle M, Angemeyer K, Hubner G and Werner S (1994) Large induction of keratinocyte growth factor expression by serum growth factors and pro-inflammatory cytokines in cultured fibroblasts. *Oncogene* **9**: 3199–3204

Chirgwin SM, Przybyla AE, Macdonald RJ and Rutter WJ (1979) Isolation of biologically active ribonucleic acid from sources enriched in ribonucleases. *Biochemistry* **18**: 5294–5299

Chomczynski P and Saachi N (1987) Single-step method of RNA extraction by acid guanidinium thiocyanate phenol-chloroform extraction. *Anal Biochem* **162**: 156–159

Church GM and Gilbert W (1984) Genomic sequencing. *Proc Natl Acad Sci USA* **81**: 1991–1995

Cullen KJ, Lippman ME, Chow D, Hill S, Rosen N and Zwiebel AE (1992) Insulin-like growth factor II overexpression in MCF-7 cells induces phenotypic changes associated with malignant progression. *Mol Endocrinol* **6**: 91–100

Feinberg AP and Vogelstein B (1983) A technique for radiolabelling DNA restriction endonuclease fragments to high specific activity. *Anal Biochem* **132**: 6–13

Finch PW, Rubin JS, Miki T, Ron D and Aaronson SA (1989) Human KGF is FGF-related with properties of a paracrine effector of epithelial cell growth. *Science* **245**: 752–755

Finch PW, Cunha GR, Rubin JS, Wong J and Ron D (1995) Pattern of keratinocyte growth factor and keratinocyte growth factor receptor expression during mouse fetal development suggests a role in mediating morphogenetic mesochymal–epithelial interactions. *Dev Dynam* **203**: 223–240

© Cancer Research Campaign 1997

Gomm JJ, Smith J, Ryall G, Baillie R, Turnbull L and Coombes RC (1991) Localisation of basic fibroblast growth factor and transforming growth factor β1 in the human mammary gland. *Cancer Res* **51**: 4685–4692

Gomm JJ, Browne P, Coope R, Liu QY, Buluwela L and Coombes RC (1995) Isolation of pure populations of epithelial and myoepithelial cells from the normal human mammary gland using immunomagnetic separation with Dynabeads. *Anal Biochem* **226**: 91–99

Hofland LJ, Van Der Berg B, Van Eijck CHJ, Sprij DM, Van Koetsveld PM and Lamberts SWJ (1995) Role of tumour-derived fibroblasts in the growth of primary cultures of human breast cancer cells; somatostatin analogue octreotide. *Int J Cancer* **60**: 93–99

Imagawa W, Cunha GR, Young P and Nandi S (1994) Keratinocyte growth factor and acidic fibroblast growth factor are mitogens for primary cultures of mammary epithelium. *Biochem Biophys Res Commun* **204**: 1165–1169

Jaakkola S, Salmikangas P, Nylund S, Partanen J, Armstrong E, Pyronen S, Lehtovirta P and Nevanlinna H (1993) Amplification of FGFR-4 gene in human breast and gynaecological cancers. *Int J Cancer* **54**: 378–382

Jaye M, Schlessinger J and Dionne C (1992) Fibroblast growth factor receptor tyrosine kinases: molecular analysis and signal transduction. *Biochim Biophys Acta* **1135**: 185–199

Koji T, Chedid M, Rubin JS, Slaydon OD, Csaky KG, Aaronson SA and Brener RM (1994) Progesterone-dependent expression of KGF mRNA in stromal cells of the primate endometrium: KGF as a progestomedin. *J Cell Biol* **125**: 393–401

Lehtola L, Partanen J, Sistonen L, Korhonen J, Warri A, Harkonen P, Clarke R and Alitalo K (1992) Analysis of tyrosine kinase mRNAs expressed in MCF7 breast cancer cells. *Int J Cancer* **50**: 598–603

Luqmani YA, Graham M and Coombes RC (1992) Expression of basic fibroblast growth factor, FGFR-1 and FGFR-2 in normal and malignant human breast and comparison with other normal tissues. *Br J Cancer* **66**: 273–280

Luqmani YA, Mortimer C, Yiangou C, Johnston CL, Bansal GS, Sinnett D, Law M and Coombes RC (1995) Expression of 2 variant forms of fibroblast growth factor receptor 1 in human breast. *Int J Cancer* **64**: 274–279

Luqmani YA, Bansal GS, Mortimer C, Buluwela L and Coombes RC (1996) Expression of FGFR2 BEK and K-SAM mRNA variants in normal and malignant human breast. *Eur J Cancer* **32A**: 518–524

McLeskey SW, Ding IYF, Lippman ME and Kern FG (1994) MDA-MB-134 breast carcinoma cells overexpress fibroblast growth factor receptors and are growth inhibited by FGF ligands. *Cancer Res* **54**: 523–530

Miki T, Fleming TP, Bottaro DP, Rubin JS, Ron D and Aaronson SA (1991) Expression cDNA cloning of the KGF receptor by creation of a transforming autocrine loop. *Science* **251**: 72–75

Miki T, Bottaro DP, Fleming TP, Smith CL, Burgess WH, Chan AM and Aaronson SA (1992) Determination of ligand-binding specificity by alternative splicing: two distinct growth factor receptors encoded by a single gene. *Proc Natl Acad Sci USA* **89**: 246–250

Miyamoto M, Naruo K, Seko C, Matsumoto S, Kondo T and Kurokawa T (1993) Molecular cloning of a novel cytokine cDNA encoding the ninth member of the fibroblast growth factor family, which has a unique secretion property. *Mol Cell Biol* **13**: 4251–4259

Pekonen F, Nyman T and Rutanen EM (1993) Differential expression of keratinocyte growth factor and its receptor in the human uterus. *Mol Cell Endocrinol* **95**: 43–49

Penault-Llorca F, Bertucci F, Adelaide J, Parc P, Coulier F, Jacquemier J, Birnbaum D and Delapeyrier O (1995) Expression of FGF and FGF receptors in human breast cancer. *Int J Cancer* **61**: 170–176

Ryan MC, Orr DJA and Horgan K (1993) Fibroblast stimulation of breast cancer cell growth in a serum-free system. *Br J Cancer* **67**: 1268–1273

Savagner P, Valles AM, Jouanneau J, Yamada KM and Thiery JP (1994) Alternative splicing in fibroblast growth factor receptor 2 is associated with induced epithelial-mesenchymal transition in rat bladder carcinoma cells. *Mol Cell Biol* **5**: 851–862

Siddiqi I, Funatomi H, Kobrin MS, Friess H, Buchler MW and Korc M (1995) Increased expression of keratinocyte growth factor in human pancreatic cancer. *Biochem Biophys Res Commun* **215**: 309–315

Singer C, Rasmussen A, Smith HS, Lippman ME, Lynch HT and Cullen KJ (1995) Malignant breast epithelium selects for insulin-like growth factor II expression in breast stroma: evidence for paracrine function. *Cancer Res* **55**: 2448–2454

Skehan P, Storeng R, Scudiero D, Monks A, McMahon J, Vistica D, Warren JT, Bokesch H, Kenney S and Boyd MR (1990) New colorimetric cytotoxicity assay for anticancer-drug screen. *J Natl Cancer Inst* **82**: 1107–1112

Smola H, Thiekotter G and Fusenig NE (1993) Mutual induction of growth factor gene expression by epidermal–dermal cell interaction. *J Cell Biol* **122**: 417–429

Stampfer M, Hallowes RC and Hackett AJ (1980) Growth of normal human mammary cells in culture. *In vitro* **16**: 415–425

Yi ES, Bedoya AA, Lee H, Kim S, Housley RM, Aukerman SL, Tarpley JE, Starnes C, Yin S and Pierce GF (1994) Keratinocyte growth factor causes dilation of the mammary glands of mice. Interactions of keratinocyte growth factor, oestrogen and progesterone in vivo. *Am J Pathol* **145**: 1015–1022

Tanaka A, Miyamoto K, Minamino N, Takeda M, Sato B, Matsuo H and Maysumoto K (1992) Cloning and characterisation of an androgen-dependent growth of mammary carcinoma cells. *Proc Natl Acad Sci USA* **89**: 8928–8932

Van Roozendaal CEP, Van Ooijen B, Klijn JGM, Classen C, Eggermont AMM, Henzen-Logmans SC and Foekens JA (1992) Stromal influences on breast cancer cell growth. *Br J Cancer* **65**: 77–81

Wilson SE, Weng J, Chwang EL, Gollahon L, Leitch AM and Shay JW (1994) Hepatocyte growth factor (HGF), keratinocyte growth factor (KGF) and their receptors in human breast cells and tissues: alternative receptors. *Cell Mol Biol Res* **40**: 337–350

Yan G, Fukabori Y, Nikolaropoulos S, Wang F and McKeehan WL (1992) Heparin-binding keratinocyte growth factor is a candidate stromal to epithelial cell andromedin. *Mol Endocrinol* **6**: 2123–2128

Yayon A, Zimmer Y, Shen GH, Avivi A, Yarden Y and Givol D (1992) A confined variable region confers ligand specificity on fibroblast growth factor receptors: implications for the origin of the immunoglobulin fold. *EMBO J* **11**: 1885–1890

Yiangou C, Johnston CL, Luqmani YA, Coope RC, Gomm JJ, Law M, Shousha S and Coombes RC (1997) Fibroblast growth factor 2 in breast cancer: occurrence and prognostic significance. *Br J Cancer* **75**: 28–33

 © Cancer Research Campaign 1997